# Supplementary material for: Candida albicans Morphology-Dependent Host FGF-2 Response as a Potential Therapeutic Target
Source: J Fungi (Basel). 2019 Mar 5;5(1):22. doi: 10.3390/jof5010022 (PMC6462958; doi:10.3390/jof5010022)
Supplement: Supplementary file 1 [file jof-05-00022-s001.pdf]

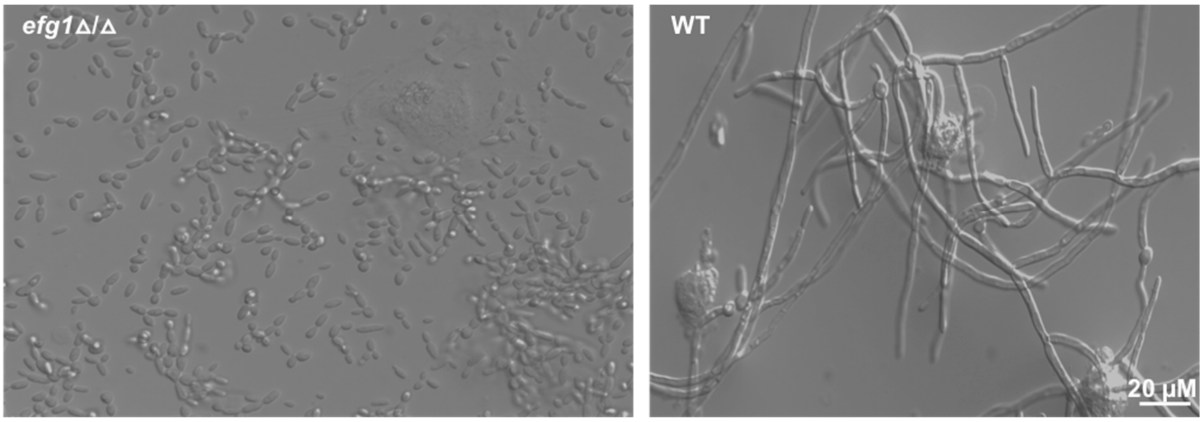

Figure S1. A comparison of *C. albicans* *efg1*Δ/Δ and WT (SC5314) morphology in the presence of host endothelial cells.

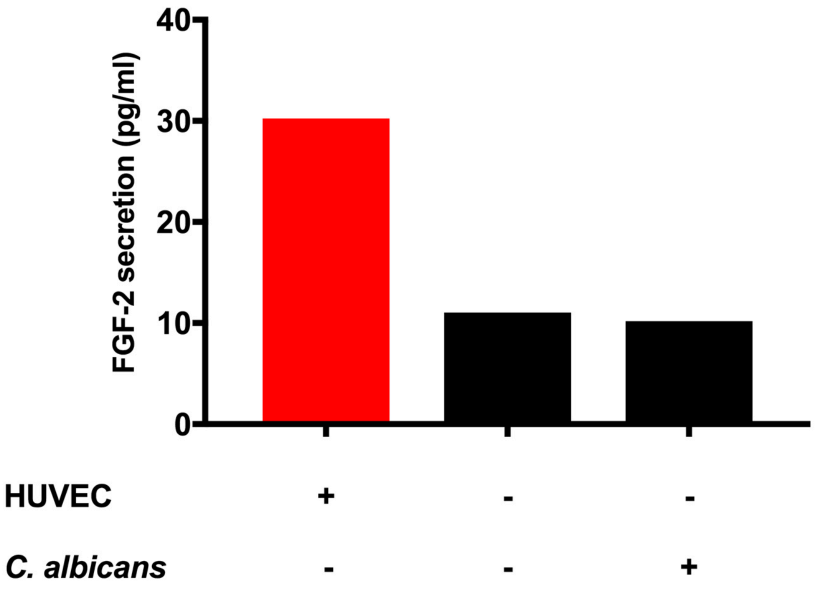

Figure S2. Comparison of FGF-2 expression between HUVEC only, medium only, and *C. albicans* only. No difference was observed between medium only and *C. albicans* only groups.

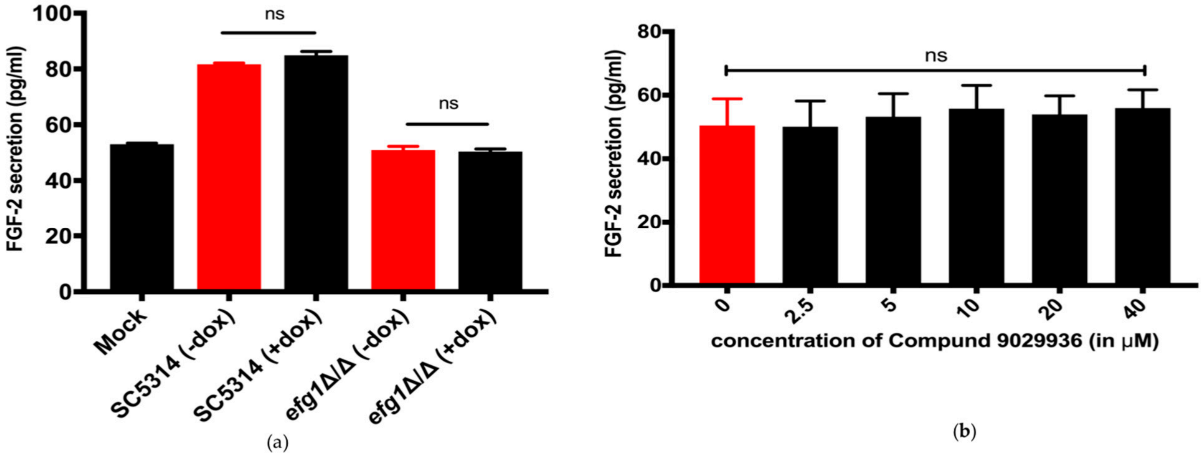

(a) (b)

Figure S3. Neither doxycycline nor compound 9029936 itself affect HUVEC FGF-2 response. a) HUVECs were challenged with wild-type strain SC5314 or *efg1*Δ/Δ (which does not have *tet* regulatable gene) in the absence or presence of doxycycline for 24 hours before measuring FGF-2 levels using ELISA. One-way ANOVA was significant ($P < 0.0001$). Tukey's multiple comparison test noted no significant difference between +dox and -dox groups of both SC5314 and *efg1*Δ/Δ. b) HUVECs were treated with 0 to 40 μM of compound 9029936 for 24 hours and the FGF-2 levels were measured in the supernatants. One-way ANOVA was not significant ($P = 0.98$).

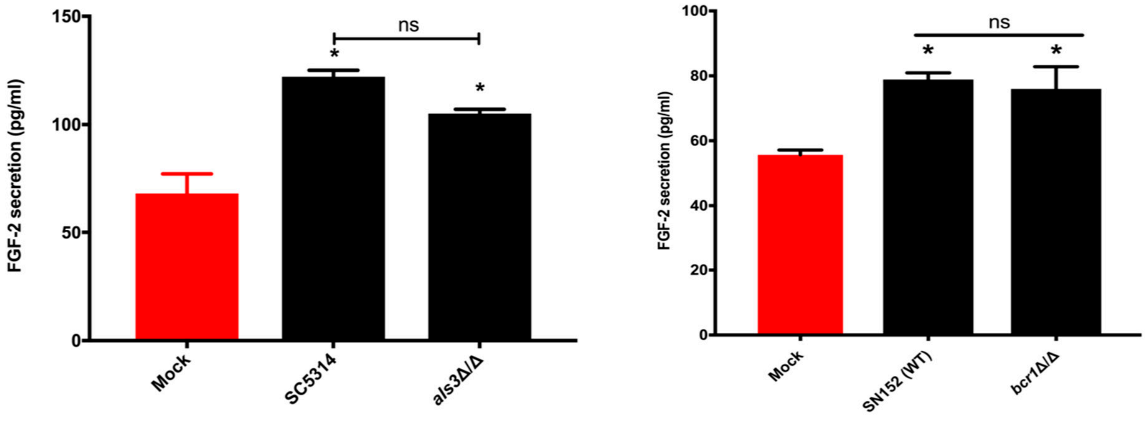

(a) (**b**)

Figure S4. ***ALS3*** **and** ***BCR1*****-regulated cell wall proteins are not involved in the regulation of host FGF-2 secretion.** a) HUVECs were challenged with either SC5314 or *als3*Δ/Δ. *C. albicans* strains for 24 hours. FGF-2 levels were measured using ELISA. While one-way ANOVA (P = 0.0002) and Dunnett's multiple comparison demonstrated a statistically significant difference (P < 0.05) between the mock and SC5314/*als3*Δ/Δ infected groups, (as indicated by *), there was no significant difference between the SC5314 and *als3*△/△ (P = 0.134) groups. b) HUVECs were challenged with either the *bcr1*△/△ mutant or SN152 parental strain for 24 hours. FGF-2 levels were measured using ELISA. Again, one-way ANOVA (P = 0.015) and Dunnett's multiple comparison (P < 0.05) only showed a statistically significant difference between mock and *C. albicans* challenged groups, (indicated by *). There was no significant difference between the SN152 parental strain and *bcr1*Δ/Δ mutant challenged groups (P = 0.134).

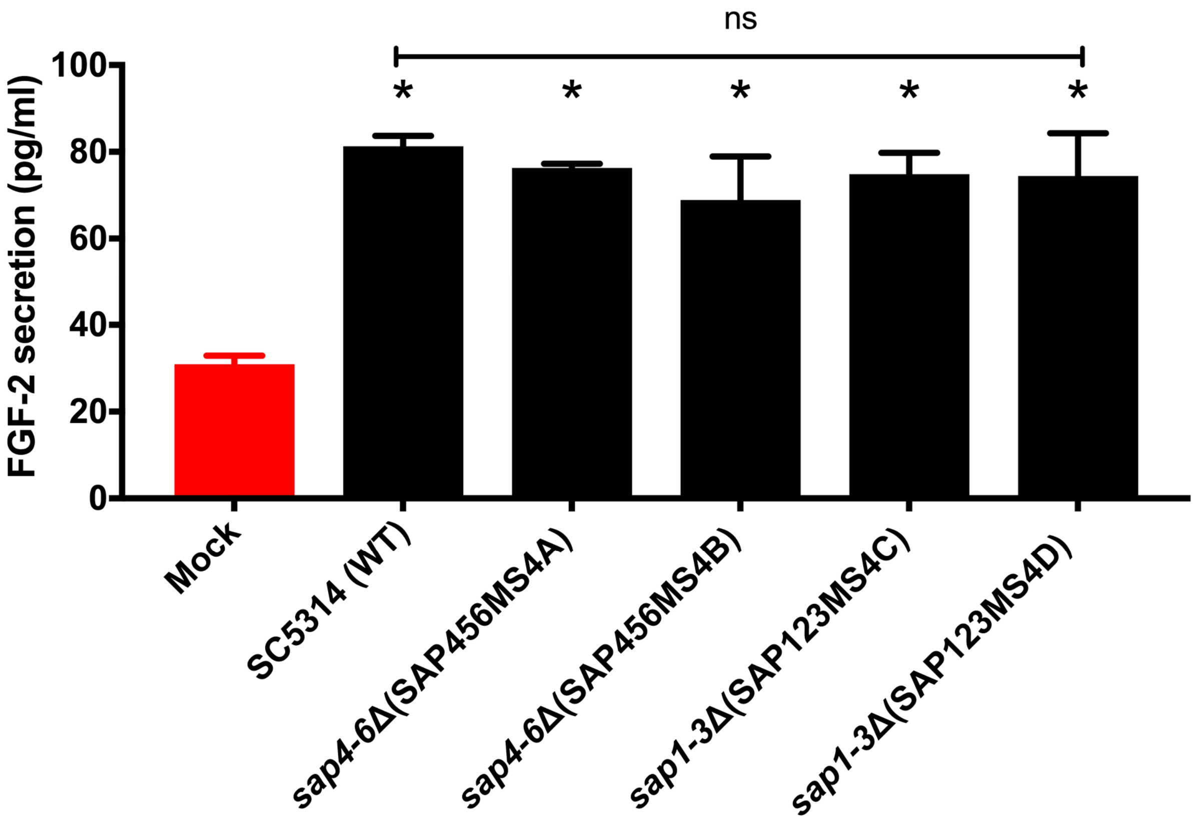

Figure S5. Sap proteins are not linked with host endothelial FGF-2 response. HUVECs were challenged with either SC5314 (WT) or the *sap1-3*Δ/Δ or *sap4-6*Δ/Δ mutant strains. Both one-way ANOVA ($P = 0.0008$) and Dunnett's multiple comparison ($P < 0.05$) demonstrated a statistically significant difference between the mock and all *C. albicans* infected groups, as indicated by (*). However, no significant difference was observed between SC5314 wild-type and *sap1-3*Δ/Δ/*sap4-6*Δ/Δ mutant infected groups ($P > 0.05$).

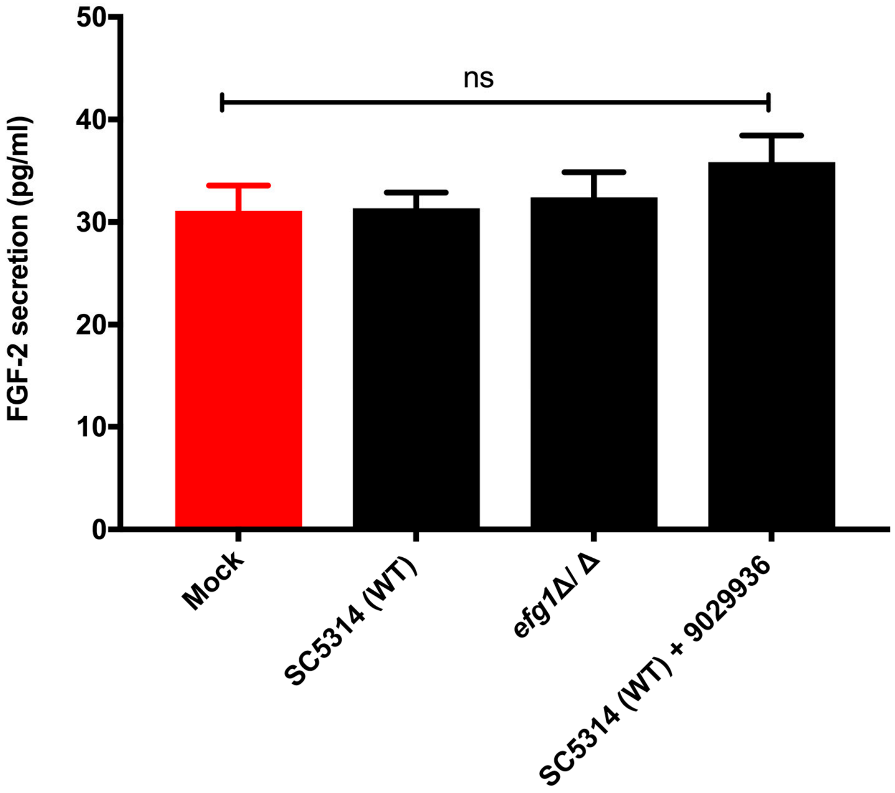

Figure S6. ***C. albicans*** **spent medium is not sufficient to increase host FGF-2 response.** *C. albicans* SC5314, *efg1*Δ/Δ or SC5314 + Compound 9029936 (10 μM) were grown in EBM at 37°C for 24 hours. The spent medium was filtered and added to HUVEC cultures for 24 hours. One-way ANOVA displayed no statistically significant difference in FGF-2 secretion between the groups ($P = 0.287$).
